# Supplementary material for: Platelet-derived chemokines promote skeletal muscle regeneration by guiding neutrophil recruitment to injured muscles
Source: Nat Commun. 2023 May 22;14:2900. doi: 10.1038/s41467-023-38624-0 (PMC10203137; doi:10.1038/s41467-023-38624-0)
Supplement: Supplementary file 1 — Supplementary Information [file 41467_2023_38624_MOESM1_ESM.pdf]

SUPPLEMENTARY FIGURES AND FIGURE LEGENDS

Platelet-derived chemokines promote skeletal muscle regeneration by guiding neutrophil recruitment to injured muscles

Flavia A. Graca, Anna Stephan, Benjamin A. Minden-Birkenmaier, Abbas Shirinifard, Yong-Dong Wang, Fabio Demontis, and Myriam Labelle

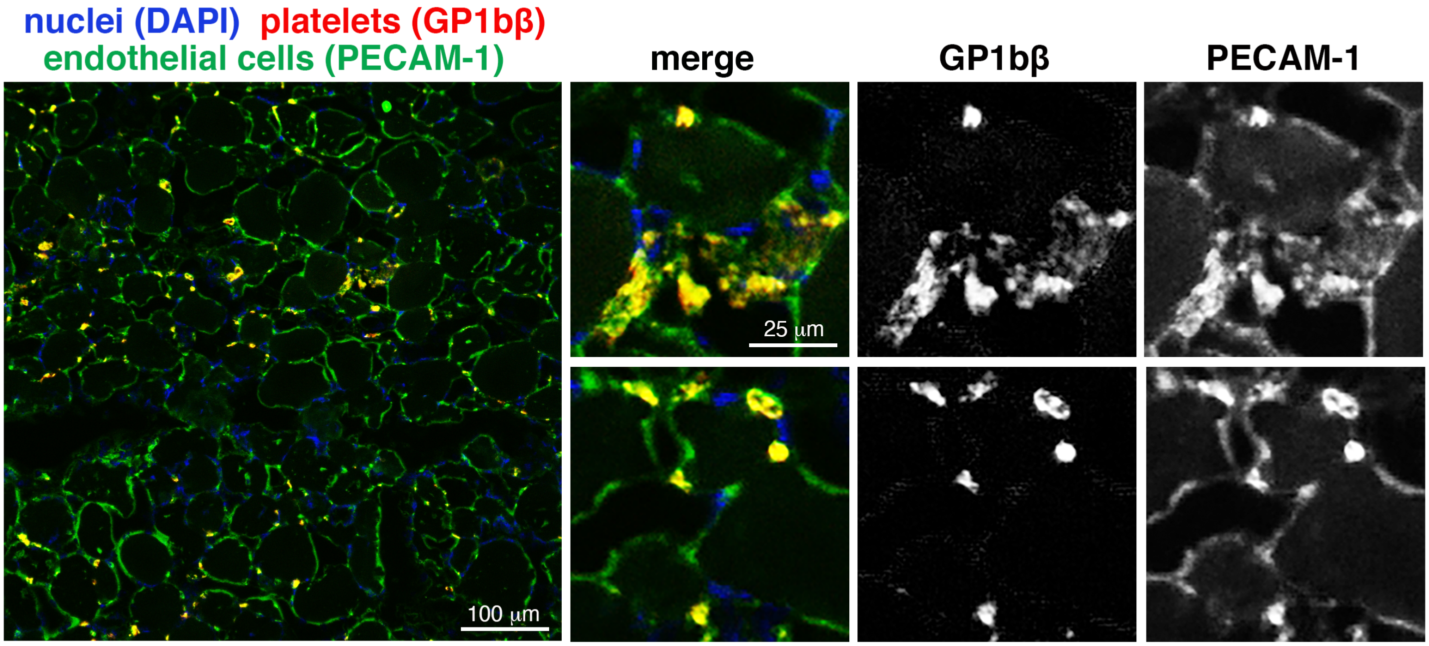

Supplementary Figure 1. Platelet clusters partially co-localize with endothelial cells in injured skeletal muscles. Immunostaining with anti-PECAM-1 antibodies (green) identifies endothelial cells of the blood vessels of skeletal muscles at day 1 after injury whereas staining with DAPI (blue) outlines the nuclei. Platelet clusters (red) are identified by immunostaining with anti-GP1bβ antibodies and co-localize (yellow) with endothelial cells of injured muscles. Representative images from 2 independent experiments. Scale bars: 100 μm (overview) and 25 μm (inset).

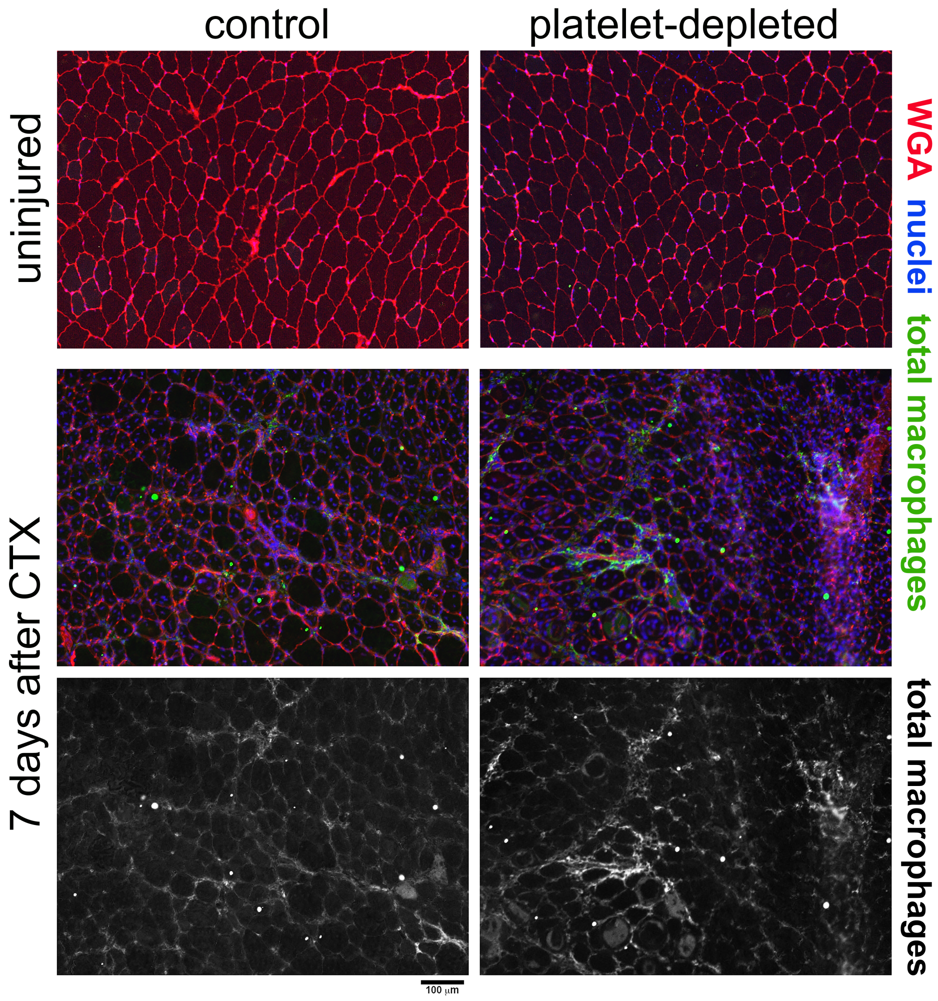

Supplementary Figure 2. Platelet depletion increases the levels of total macrophages that infiltrate injured skeletal muscles at day 7 from cardiotoxin injection. Immunostaining with anti-F4/80 antibodies identifies infiltrating macrophages (green) in regenerating skeletal muscles at day 7 after injury. Staining with WGA (red) identifies the myofiber boundaries. As expected based on previous studies, no substantial presence of macrophages is detected in uninjured skeletal muscles (from control and platelet-depleted mice). Macrophages are detected in skeletal muscles at day 7 from cardiotoxin (CTX) injection, and there is increased macrophage infiltration in muscles from platelet-depleted mice. Scale bar, 100 μm. Representative images from 2 independent experiments. See also data in Fig. 2f.

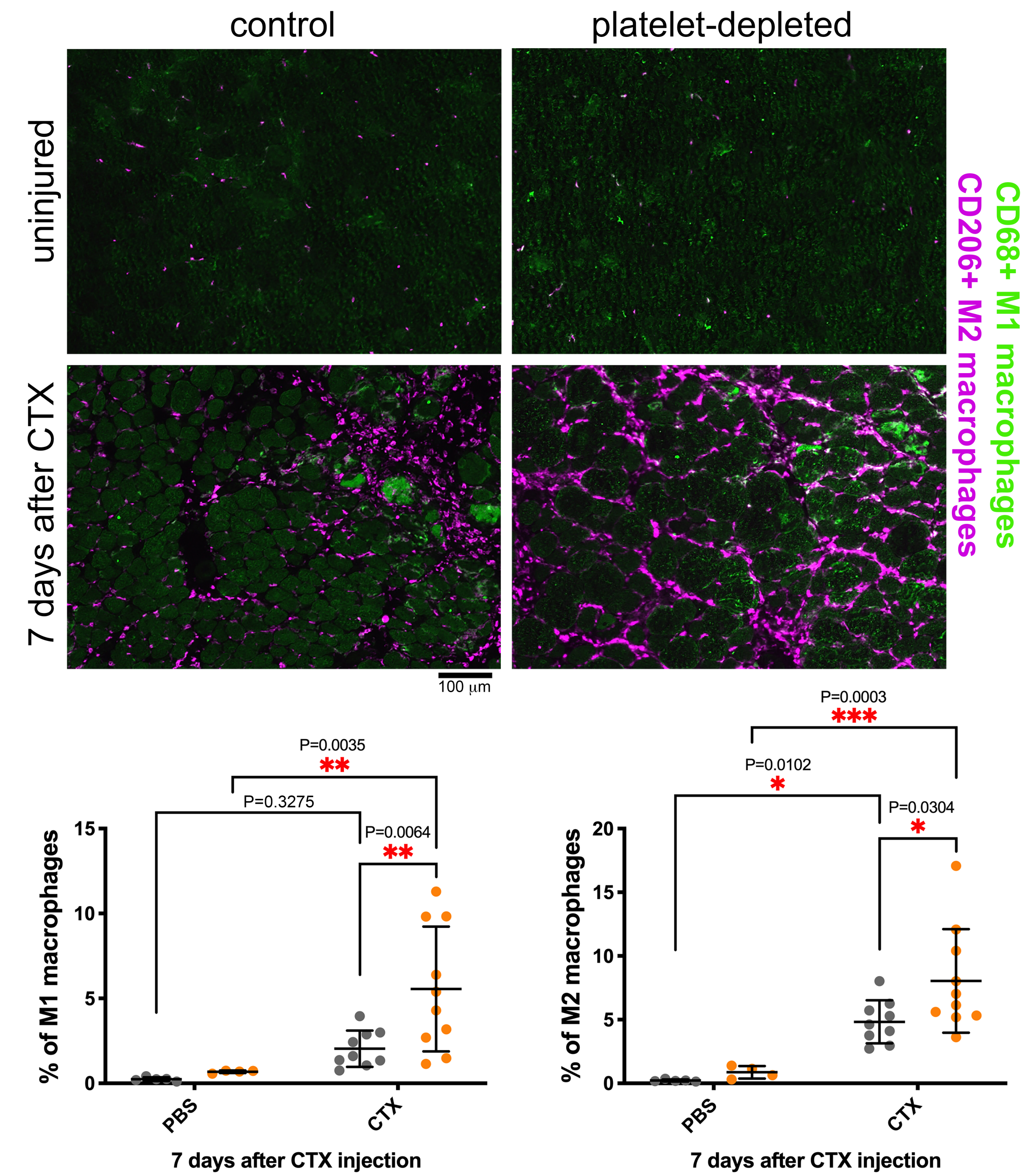

Supplementary Figure 3. Platelet depletion increases the levels of muscle-infiltrating M1 and M2 macrophages. Immunostaining with anti-CD68 and anti-CD206 antibodies identifies M1 and M2 macrophages (respectively in green and purple) in regenerating skeletal muscles. The levels of muscle-infiltrating M1 and M2 macrophages increase at day 7 from cardiotoxin (CTX) injection compared to uninjured controls. Such infiltration is higher in muscles from platelet-depleted versus control mice. Scale bar, 100 µm. The graph displays the mean ±SD, with n(PBS-control)=5, n(CTX-control)=9, n(PBS-platelet depleted)=4, and n(CTX-platelet depleted)=10 biologically independent muscles obtained from the same number of independent mice; *P<0.05, **P<0.01, ***P<0.001 (two-way ANOVA with Sidak post-hoc test). Source data are provided in the Source Data file.

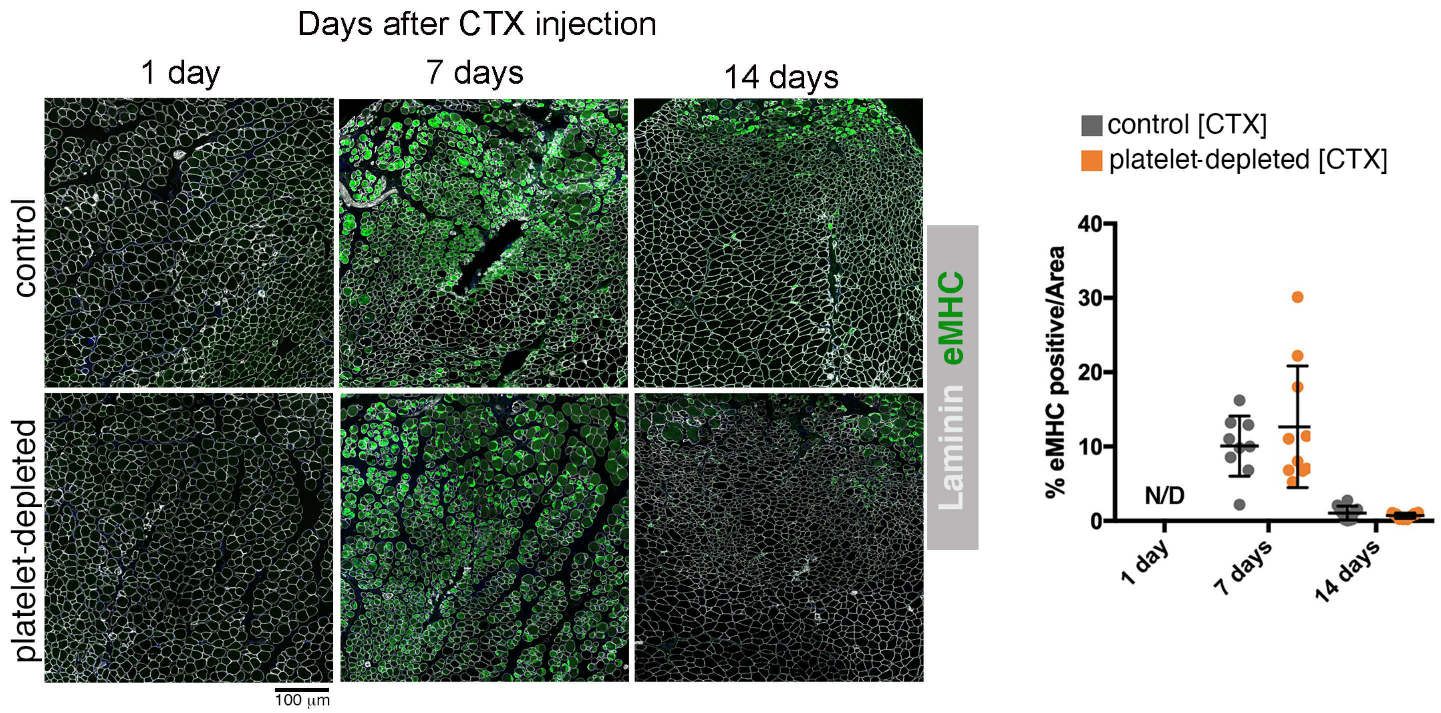

Supplementary Figure 4. Similar levels of eMHC are found in regenerating skeletal muscles from control and platelet-depleted mice. Immunodetection of embryonic myosin heavy chain (eMHC) in regenerating TA skeletal muscles from platelet-depleted and control mice. Normally, eMHC is expressed during muscle development and disappears after birth but it is re-expressed during myogenesis associated with muscle regeneration. Consistently, there are many eMHC-positive myofibers at day 7 after cardiotoxin-induced injury, coincident with myogenesis, but their abundance declines at day 14 post-injury, a day at which myogenesis and muscle regeneration are largely resolved. Substantially similar percentages of eMHC-positive tissue areas are found in regenerating skeletal muscles from control and platelet-depleted mice. eMHC is not detected (N/D) at day 1 after injury. Scale bar, 100 μm. The graph displays the mean ±SD with n(control CTX day 7)=9 and n=10 for all other groups. Each n corresponds to a biologically independent muscle sourced from an independent mouse. Statistical analysis was done with two-way ANOVA with Tukey post-hoc test. Source data are provided in the Source Data file.

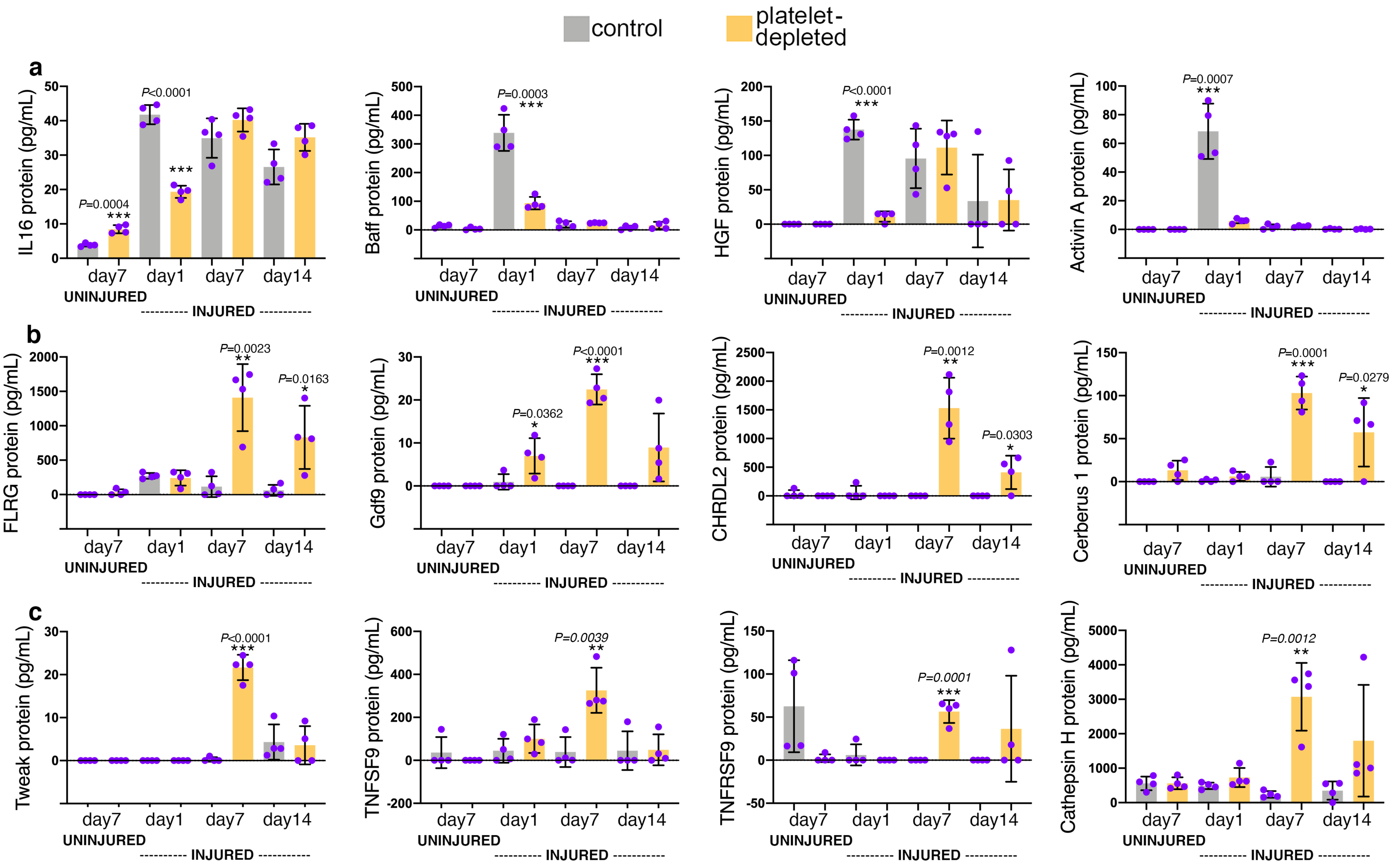

Supplementary Figure 5. Additional examples of cytokines and growth factors that are differentially regulated in regenerating skeletal muscles from platelet-depleted and control mice. Cytokine concentrations in muscle homogenates from control (grey) and platelet-depleted mice (yellow).

a IL16 is a T-lymphocyte chemoattractant factor [1] which was upregulated in uninjured muscles but downregulated at day 1 after injury in muscles from platelet-depleted mice. Similarly, Baff (a TNF cytokine), Hgf (hepatocyte growth factor), and Activin A (a TGF-β ligand) were upregulated at day 1 after injury but less so in muscles from platelet-depleted mice. Modulation of these proteins by platelet depletion likely influences muscle regeneration [2, 3, 4, 5]. However, apart from IL16 (which can be secreted by platelets [6]), decline in their levels upon platelet depletion is not directly due to platelets as these cytokines are not detected in the platelet proteome [6]: muscle cells or infiltrating immune cells may modulate their levels in regenerating muscles.

b Other secreted factors that increase upon platelet depletion include Flrg/Fstl3 (Follistatin-like 3), Gdf9, Chrdl2 (which binds TGF-β), and Cerberus 1 (a BMP antagonist).

c The TNF ligands Tweak (TNFSF12) and 4-1BBL (TNFSF9) increase in muscles from platelet-depleted mice at day 7 from injury. The TNF receptor 4-1BB (TNFRSF9) is similarly upregulated. Although tumor necrosis factor receptor signaling promotes myogenesis during muscle regeneration [7, 8, 9, 10], this pathway can stunt myofiber growth and induce myofiber atrophy [11, 12, 13, 14]. Therefore, TNF upregulation in regenerating muscles of platelet-depleted mice likely contributes to the reduced myofiber size that is found in these muscles post-injury. Moreover, cathepsin H is also upregulated in the muscles of platelet-depleted mice and this lysosomal enzyme may contribute to myofiber atrophy by promoting proteolysis [13, 14, 15].

The graphs display the mean ±SD, with n=4 biologically independent muscles from 4 independent mice for each timepoint and condition; *$P<0.05$, **$P<0.01$, ***$P<0.001$ (unpaired two-tailed t-test) refer to the comparison of muscles from control versus platelet-depleted mice at each day of regeneration.
Source data are provided in the Source Data file.

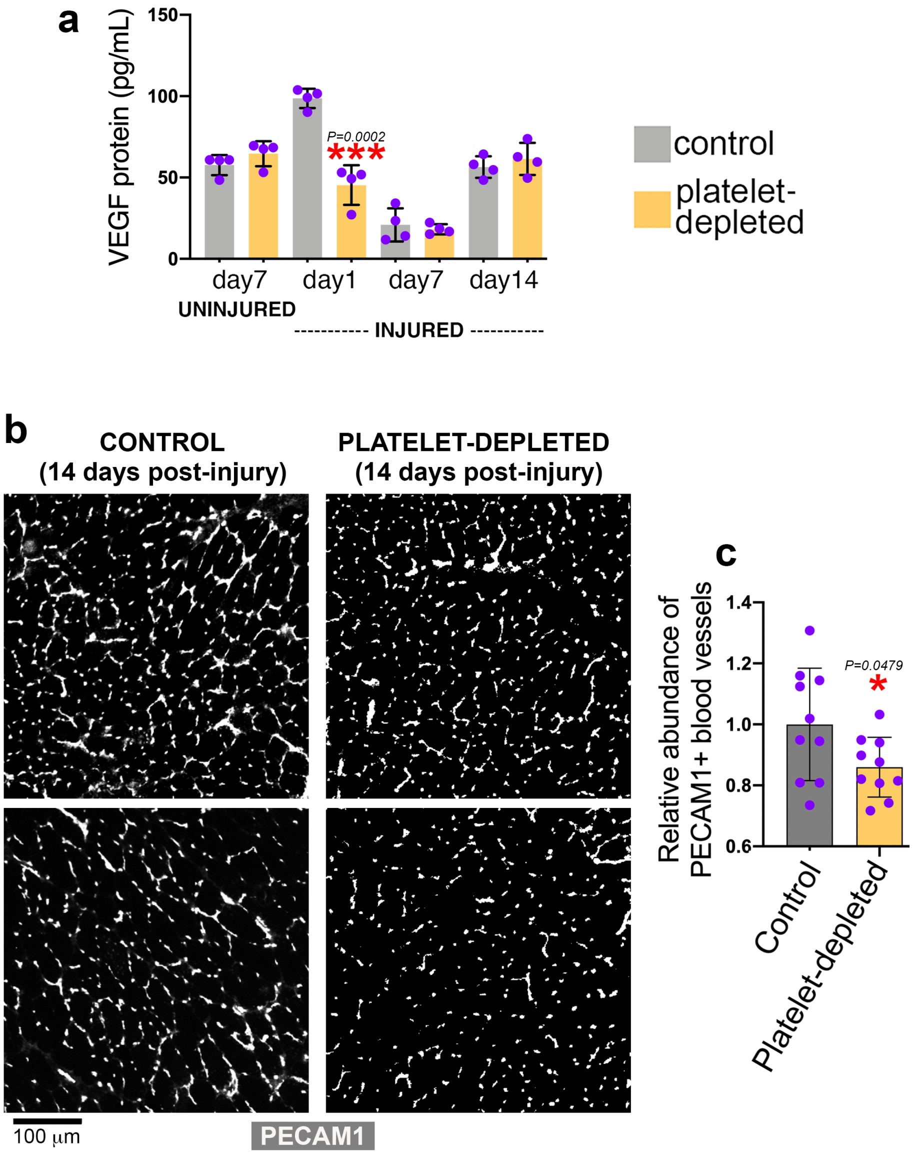

Supplementary Figure 6. Platelet depletion impairs neo-angiogenesis in regenerating skeletal muscles. **a** Platelet depletion reduces the intramuscular levels of the pro-angiogenic factor VEGF in the early phase of muscle regeneration. VEGF protein levels peak at day 1 after injury, which corresponds to the time of neutrophil infiltration to injured muscles, but VEGF levels are reduced by platelet depletion. The graphs display the mean ±SD with n=4 biologically independent muscles from 4 independent mice for each timepoint and condition; ***P<0.001 (unpaired two-tailed t-test). **b-c** Platelet depletion decreases blood vessel density in post-injury skeletal muscles. Immunostaining with anti-PECAM1 antibodies (white) of TA muscle cross-sections indicates that there is a decline in blood vessel density in post-injury TA muscles (14 days from CTX) from platelet-depleted versus control mice. Scale bar, 100 μm. In (c), the graph displays the mean ±SD with n=10 biologically independent muscle samples obtained from n=10 independent mice for each group; *P<0.05 (unpaired two-tailed t-test). Source data are provided in the Source Data file.

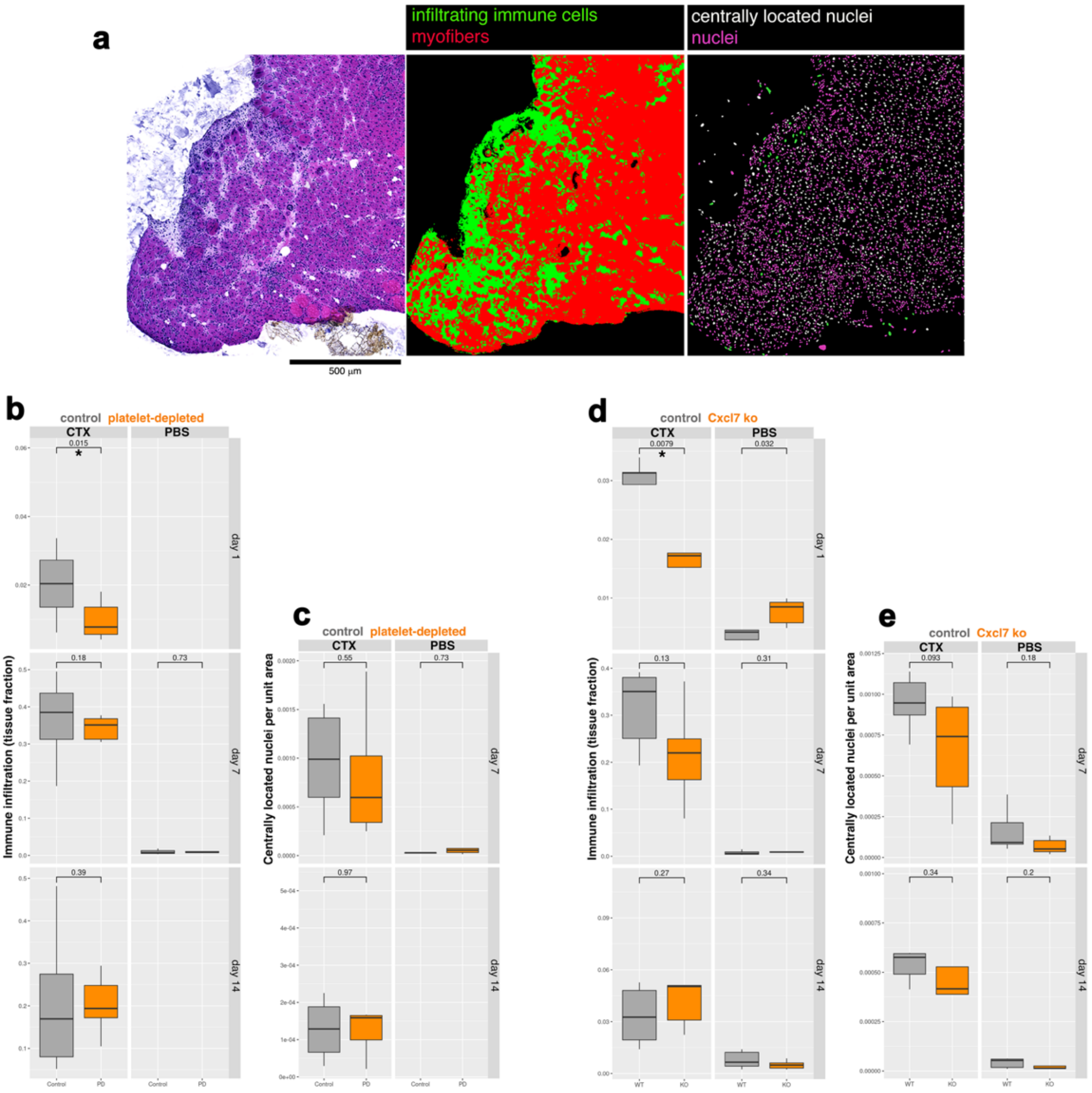

Supplementary Figure 7. Automated image analysis identifies defective immune infiltration as an early step of muscle regeneration that is consistently altered by platelet depletion and Cxcl7 knockout.
a Identification of muscle-infiltrating immune cells, myofibers, and centrally-nucleated myofibers via automated analysis of H&E images. Scale bar, 500 μm. **b** There is a significant decrease in immune infiltration in the TA muscles from platelet-depleted mice at day 1 after CTX-induced injury, compared to controls. No significant changes in immune infiltration are found at day 7 and day 14 after injury. Minimal immune infiltration is found in uninjured (PBS-injected) TA muscles. **c** Myofibers with central nuclei are primarily detected after 7 days from CTX-mediated injury but not in uninjured muscles. There are no significant changes in the number of centrally-nucleated myofibers when comparing platelet-depleted versus control TA muscles. **d** As observed for muscles from platelet-depleted mice, a significant decline in immune infiltration is found in muscles from Cxcl7ko mice at day 1 after CTX injection, compared to injured muscles from littermate wild-type controls. **e** There are no significant changes in the number of centrally-nucleated myofibers detected in injured muscles from Cxcl7ko versus control mice. In these graphs, the center line is the median whereas the lower and upper hinges correspond to the first and third quartiles (the 25th and 75th percentiles). The upper whisker extends from the hinge to the largest value, no further than 1.5 * IQR (inter-quartile range) from the hinge. The lower whisker extends from the hinge to the smallest value, at most 1.5 * IQR from the hinge; n=5 images of biologically independent muscles obtained from n=5 independent mice; *P<0.05 (two-way Mann-Whitney U test). Source data are provided in the Source Data file.

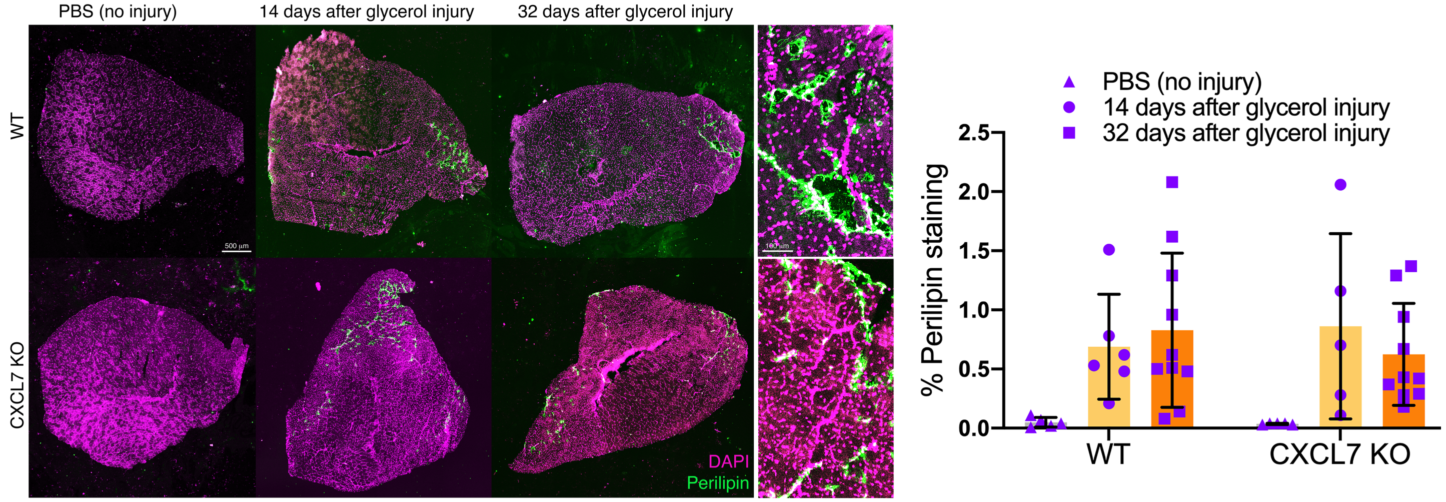

Supplementary Figure 8. Muscle fat infiltration occurs similarly in post-injury TA skeletal muscles from control and Cxcl7ko mice. Analysis of control (uninjured) and glycerol-injured tibialis anterior skeletal muscles at 14 and 32 days after damage. Immunostaining with anti-Perilipin-1 antibodies (green) is used to identify fat infiltrations in post-injury muscles whereas staining with DAPI (purple) identifies the nuclei. As expected, there is a striking increase in the muscle fatty infiltration after glycerol-induced injury but this occurs similarly in the muscles of Cxcl7ko versus control mice. Scale bars: 500 μm (muscle overview) and 100 μm (inset).

The graph displays the mean ±SD with n(PBS-no injury, WT & Cxcl7ko)=5, n(WT-14 days from CTX)=6, n(Cxcl7ko-14 days from CTX)=5, n(32 days from CTX, WT & Cxcl7ko)=10 biologically independent muscles obtained from the same number of independent mice. Statistical analysis was done with two-way ANOVA with Tukey post-hoc test. Source data are provided in the Source Data file.

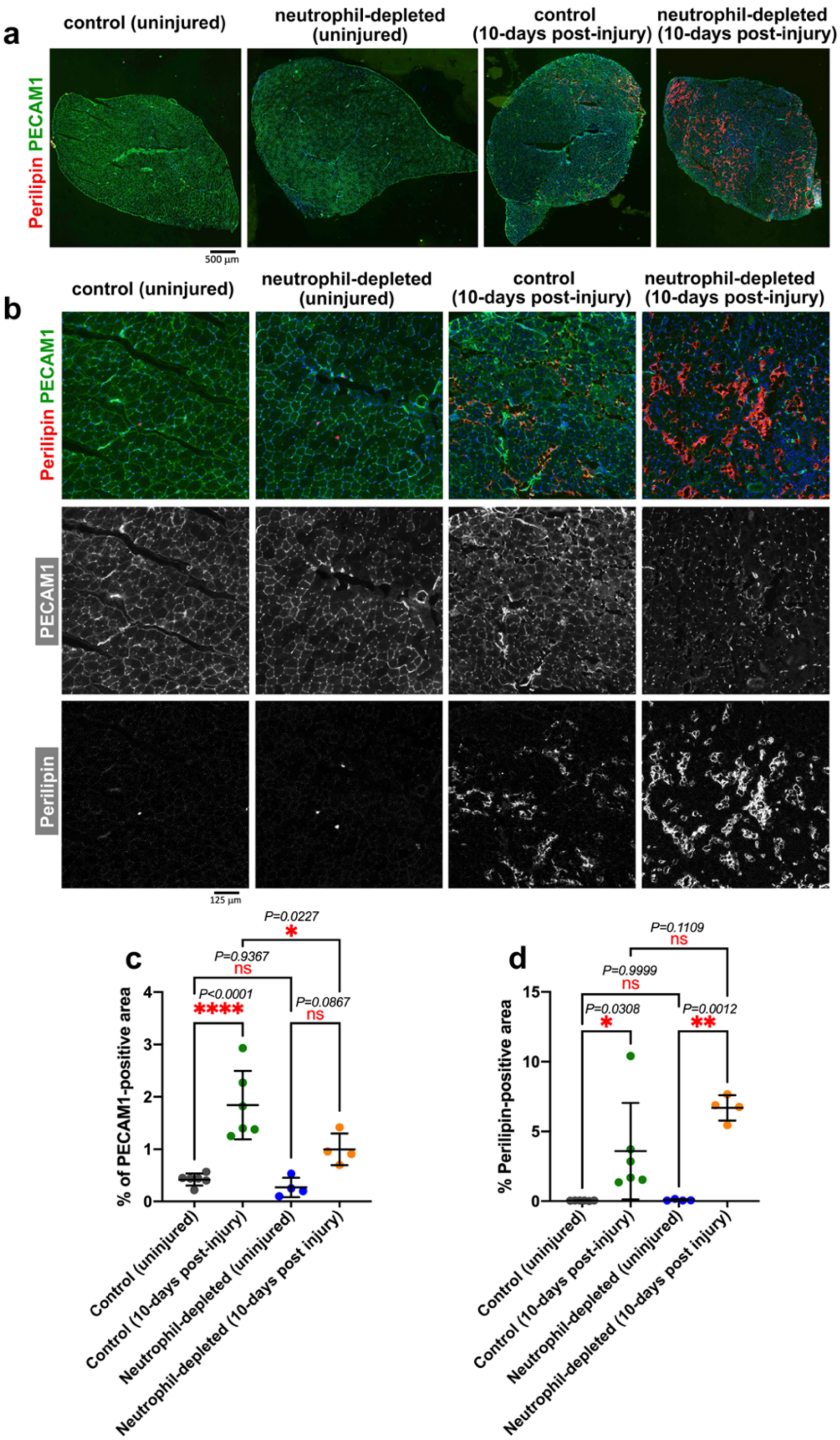

Supplementary Figure 9. Neutrophil depletion significantly impairs neo-angiogenesis and partially increases fat infiltration. a-b Analysis of control (uninjured) and glycerol-injured tibialis anterior skeletal muscles at 10 days after damage. Immunostaining with anti-Perilipin-1 antibodies (red) is used to identify fat infiltrations whereas staining with PECAM1 (green) identifies the capillaries. Scale bars: 500 μm (a) and 125 μm (b). **c** Neutrophil depletion reduces PECAM1 immunoreactivity, suggesting that the neo-angiogenesis associated with regeneration is impaired. **d** There is a trend towards increased muscle fat infiltration after injury in the muscles of mice that are neutrophil-depleted versus controls. The graphs in c-d display the mean ±SD, with n(from control mice)=6 and n(from neutrophil-depleted mice)=4 biologically independent muscles sourced from the same number of independent mice; *P<0.05, **P<0.01, ***P<0.001, ns=not significant (two-way ANOVA with Tukey post-hoc test). Source data are provided in the Source Data file.

SUPPLEMENTARY REFERENCES

1. Laberge S, Cruikshank WW, Beer DJ, Center DM. Secretion of IL-16 (lymphocyte chemoattractant factor) from serotonin-stimulated CD8+ T cells in vitro. *J Immunol* **156**, 310-315 (1996).

2. Latres E*, et al.* Activin A more prominently regulates muscle mass in primates than does GDF8. *Nat Commun* **8**, 15153 (2017).

3. Yaden BC*, et al.* Inhibition of activin A ameliorates skeletal muscle injury and rescues contractile properties by inducing efficient remodeling in female mice. *Am J Pathol* **184**, 1152-1166 (2014).

4. Krystufkova O*, et al.* Expression of BAFF receptors in muscle tissue of myositis patients with anti-Jo-1 or anti-Ro52/anti-Ro60 autoantibodies. *Arthritis Res Ther* **16**, 454 (2014).

5. Choi W, Lee J, Lee J, Lee SH, Kim S. Hepatocyte Growth Factor Regulates Macrophage Transition to the M2 Phenotype and Promotes Murine Skeletal Muscle Regeneration. *Front Physiol* **10**, 914 (2019).

6. Zeiler M, Moser M, Mann M. Copy number analysis of the murine platelet proteome spanning the complete abundance range. *Mol Cell Proteomics* **13**, 3435-3445 (2014).

7. Girgenrath M*, et al.* TWEAK, via its receptor Fn14, is a novel regulator of mesenchymal progenitor cells and skeletal muscle regeneration. *EMBO J* **25**, 5826-5839 (2006).

8. Enwere EK, Lacasse EC, Adam NJ, Korneluk RG. Role of the TWEAK-Fn14-cIAP1-NF-kappaB Signaling Axis in the Regulation of Myogenesis and Muscle Homeostasis. *Front Immunol* **5**, 34 (2014).

9. Shcherbina A*, et al.* Dissecting Murine Muscle Stem Cell Aging through Regeneration Using Integrative Genomic Analysis. *Cell Rep* **32**, 107964 (2020).

10. Chen SE, Jin B, Li YP. TNF-alpha regulates myogenesis and muscle regeneration by activating p38 MAPK. *Am J Physiol Cell Physiol* **292**, C1660-1671 (2007).

11. De Larichaudy J*, et al.* TNF-alpha- and tumor-induced skeletal muscle atrophy involves sphingolipid metabolism. *Skelet Muscle* **2**, 2 (2012).

12. Reid MB, Li YP. Tumor necrosis factor-alpha and muscle wasting: a cellular perspective. *Respir Res* **2**, 269-272 (2001).

13. Piccirillo R, Demontis F, Perrimon N, Goldberg AL. Mechanisms of muscle growth and atrophy in mammals and Drosophila. *Dev Dyn* **(2)** 201-215 (2014).

14. Sartori R, Romanello V, Sandri M. Mechanisms of muscle atrophy and hypertrophy: implications in health and disease. *Nat Commun* **12**, 330 (2021).

15. Lecker SH*, et al.* Multiple types of skeletal muscle atrophy involve a common program of changes in gene expression. *FASEB journal : official publication of the Federation of American Societies for Experimental Biology* **18**, 39-51 (2004).
