# Supplementary material for: Platelet-derived chemokines promote skeletal muscle regeneration by guiding neutrophil recruitment to injured muscles
Source: Nat Commun. 2023 May 22;14:2900. doi: 10.1038/s41467-023-38624-0 (PMC10203137; doi:10.1038/s41467-023-38624-0)
Supplement: Supplementary file 3 — Reporting Summary [file 41467_2023_38624_MOESM3_ESM.pdf]

nature portfolio

Corresponding author(s): Fabio Demontis and Myriam Labelle

Last updated by author(s): Apr 21, 2023

Reporting Summary

Nature Portfolio wishes to improve the reproducibility of the work that we publish. This form provides structure for consistency and transparency in reporting. For further information on Nature Portfolio policies, see our Editorial Policies and the Editorial Policy Checklist.

Statistics

For all statistical analyses, confirm that the following items are present in the figure legend, table legend, main text, or Methods section.

n/a	Confirmed	
☐	☒	The exact sample size (*n*) for each experimental group/condition, given as a discrete number and unit of measurement
☐	☒	A statement on whether measurements were taken from distinct samples or whether the same sample was measured repeatedly
☐	☒	The statistical test(s) used AND whether they are one- or two-sided<br>*Only common tests should be described solely by name; describe more complex techniques in the Methods section.*
☐	☒	A description of all covariates tested
☐	☒	A description of any assumptions or corrections, such as tests of normality and adjustment for multiple comparisons
☐	☒	A full description of the statistical parameters including central tendency (e.g. means) or other basic estimates (e.g. regression coefficient) AND variation (e.g. standard deviation) or associated estimates of uncertainty (e.g. confidence intervals)
☐	☒	For null hypothesis testing, the test statistic (e.g. *F*, *t*, *r*) with confidence intervals, effect sizes, degrees of freedom and *P* value noted<br>*Give P values as exact values whenever suitable.*
☒	☐	For Bayesian analysis, information on the choice of priors and Markov chain Monte Carlo settings
☒	☐	For hierarchical and complex designs, identification of the appropriate level for tests and full reporting of outcomes
☒	☐	Estimates of effect sizes (e.g. Cohen's *d*, Pearson's *r*), indicating how they were calculated

Our web collection on statistics for biologists contains articles on many of the points above.

Software and code

Policy information about availability of computer code

Data collection	Data collection was done with Microsoft Excel (version 14.7.3), GraphPad Prism (version 8), Nikon Elements software (version 4.11.0), and Lab-View-based DMC program (version 5.202; Aurora Scientific).
Data analysis	Analysis of immunostaining. For the analysis of immune cell infiltration, the images were threshold-adjusted and the percentage of the muscle field area occupied by immune cells was calculated by using the Nikon Elements software. All images within an experiment were processed equally. For myofiber size and type analysis, TA muscle sections were incubated with antibodies against type 2A (DSHB, cat no. SC-71) and 2B myosin heavy chain (DSHB, cat no. BF-F3) and rat anti-laminin a2 (4H8-2; Santa Cruz, cat no. sc-59854) overnight at 4°C. The sections were then washed and incubated with secondary antibodies for type 2A (anti-mouse IgG1 Alexa488), type 2B (anti-mouse IgM Alexa555), and laminin (anti-rat IgG Alexa633). The whole tibialis anterior section was imaged on a Nikon C2 confocal microscope with a 10x objective and stitched to compile an overview of the muscle. The fiber types and sizes were analyzed with the Nikon Elements software using the inverse threshold of laminin a2 staining to determine myofiber boundaries. The myosin heavy chain staining was used to classify type 2B fibers (red), type 2A (green), and presumed 2X fibers (black) that were not stained for 2B or 2A. After the myofibers were classified and the parameters measured, the Feret's minimal diameter was used as measurement of the myofiber size due to its accuracy in estimating the size of unevenly shaped or cut objects. For the quantification of the number of myofibers, all fibers in the cross-sections of entire tibialis anterior muscles were counted based on the myofiber borders identified by laminin immunostaining. The size and number of myofibers were measured from the inverse images of laminin immunostaining (for identifying myofiber borders), excluding myofibers with diameters <2 and >100µm. To categorize myofiber types, the intersections of the inverse images of laminin and myosin heavy chain-specific staining were used. These analyses were performed using the Nikon Elements software (version 4.11.0) and the "Object count" function.<br><br>H&E image analysis. H&E slides were scanned at 20x. For estimating the infiltration of immune cells into skeletal muscles, the Ilastik machine learning software was used to segment the immune infiltration regions and total muscle tissue area in each slide, which lead to quantify the

ratio of immune infiltration versus the total muscle area. For quantifying myofibers with centrally-located nuclei, we used the StarDist deep learning model to segment all the nuclei in each slide. Subsequently, the Ilastik software package was used to generate a stack of 36 features for each slide. We used the feature stacks and the segmented nuclei as an input for the Ilastik object classification workflow and trained a classifier to detect centrally located nuclei. We used the muscle segmentation and immune infiltration mask to remove the nuclei located outside the muscle tissue and the nuclei located in the immune-infiltrated muscle regions.

Analysis of results obtained with cytokine antibody arrays. TA muscle tissues were homogenized in a bullet blender at 4ºC with 0.5-mm zirconium beads and RayBio Lysis Buffer for antibody arrays (RayBio Lysis Buffer; AA-LYS-10mL) with protease inhibitors. After homogenization, the lysates were centrifuged for 5 min at 10,000 x g to remove tissue debris and the supernatant was collected and used for probing the cytokine arrays. 10 µL of the supernatant was used for protein quantitation. For each sample, 350 µL (at ~1-2 mg/mL) were applied to the Quantibody Mouse Cytokine Antibody Array 640 (RayBiotech, catalog #: QAM-CAA-640), a combination of 16 non-overlapping antibody arrays to quantitatively measure 640 mouse cytokines, and processed by the manufacturer according to the standard procedures listed in the manual for this product. The final concentration of each target cytokine (pg/mL) in each sample was utilized for hierarchical clustering and to generate a heatmap. Specifically, the cytokine heatmap was generated from z-scores of cytokine protein levels, after assigning a base value to each cytokine using 2 x Z-score (min non-zero), which was used to replace missing values, i.e. concentrations of 0 pg/mL. Subsequently, a clustering method of UGPMA (unweighted pair group method with arithmetic mean) and similarity measure of correlation were applied, using the Spotfire (v7.5.0, TIBCO) Hierarchical Clustering tool.

Analysis of the muscle force data was done by using a Lab-View-based DMC program (version 5.202; Aurora Scientific).

For manuscripts utilizing custom algorithms or software that are central to the research but not yet described in published literature, software must be made available to editors and reviewers. We strongly encourage code deposition in a community repository (e.g. GitHub). See the Nature Portfolio guidelines for submitting code & software for further information.

Data

Policy information about availability of data

All manuscripts must include a data availability statement. This statement should provide the following information, where applicable:

- Accession codes, unique identifiers, or web links for publicly available datasets
- A description of any restrictions on data availability
- For clinical datasets or third party data, please ensure that the statement adheres to our policy

All the primary data corresponding to the figures and supplementary figures of this study are available in the Source Data File.

Human research participants

Policy information about studies involving human research participants and Sex and Gender in Research.

Reporting on sex and gender	N/A
Population characteristics	N/A
Recruitment	N/A
Ethics oversight	N/A

Note that full information on the approval of the study protocol must also be provided in the manuscript.

Field-specific reporting

Please select the one below that is the best fit for your research. If you are not sure, read the appropriate sections before making your selection.

☒ Life sciences ☐ Behavioural & social sciences ☐ Ecological, evolutionary & environmental sciences

For a reference copy of the document with all sections, see nature.com/documents/nr-reporting-summary-flat.pdf

Life sciences study design

All studies must disclose on these points even when the disclosure is negative.

Sample size	Sample size was determined in accordance to standard practices in this field of research and based on previous analyses and experience with this or similar experimental paradigms (PMID: 26584623; PMID: 35501350; PMID: 33658508).
Data exclusions	No data points were excluded.
Replication	Mouse studies were not replicated but they included sufficient numbers to account for biological variability. Moreover, multiple techniques and models were used to validate the same findings, each with 4 or more biological replicates.

Randomization	The samples/animals were allocated to groups randomly to reduce the chance of batching effects.
Blinding	The samples and different conditions were not processed in a blinded manner by the researchers participating in this study because they rely on objective instrument measurements. Moreover, image analysis and quantitation was done in an automated (and hence blinded) manner. All experiments were reviewed by 2-3 independent investigators.

Reporting for specific materials, systems and methods

We require information from authors about some types of materials, experimental systems and methods used in many studies. Here, indicate whether each material, system or method listed is relevant to your study. If you are not sure if a list item applies to your research, read the appropriate section before selecting a response.

Materials & experimental systems

n/a	Involved in the study
☐	☒ Antibodies
☒	☐ Eukaryotic cell lines
☒	☐ Palaeontology and archaeology
☐	☒ Animals and other organisms
☒	☐ Clinical data
☒	☐ Dual use research of concern

Methods

n/a	Involved in the study
☒	☐ ChIP-seq
☒	☐ Flow cytometry
☒	☐ MRI-based neuroimaging

Antibodies

Antibodies used	For immunostaining muscle-infiltrating immune cells, the following antibodies were used: anti-MMP9 (R&D Systems, cat. no. AF909) and anti-Ly6G (BioLegend, cat. no. 17-9668-82) to detect neutrophils; anti-F4/80 (BioLegend; cat. no. 123119) to immunostain total macrophages, anti-CD68 to immunostain M1 macrophages (Abcam, cat. no. ab125212), and anti-CD206 (Macrophage Mannose Receptor; 6068c2, Biolegend, cat. no. 141711) to immunostain M2 macrophages; and anti-GP1bb antibodies to detect platelets (Emfret, cat. no. X649). In addition, rat anti-laminin a2 antibodies (4H8-2; Santa Cruz, cat. no. sc-59854) or WGA (Wheat Germ Agglutinin, Alexa Fluor 555 conjugate, ThermoFisher, cat. no. W32464) were used to delineate the myofiber boundaries. Anti-eMHC antibodies were used to detect embryonic myosin heavy chain (anti-MYH3, Santa Cruz, cat. no. SC-5309). Anti-PECAM-1 antibodies (MEC13.3; BD Biosciences) were utilized to identify blood vessels. Immunostaining for Perilipin-1 (Cell Signaling Technologies, cat. no. 9349) was used to identify fat infiltration in skeletal muscles after regeneration. Nuclei were detected with DAPI (4',6-diamidino-2-phenylindole; Roche, cat. no. 10236276001).<br>For myofiber size and type analysis, TA muscle sections were incubated with antibodies against type 2A (DSHB, cat. no. SC-71) and 2B myosin heavy chain (DSHB, cat. no. BF-F3) and rat anti-laminin a2 (4H8-2; Santa Cruz, cat. no. sc-59854) overnight at 4°C. The sections were then washed and incubated with secondary antibodies for type 2A (anti-mouse IgG1 Alexa488, Life Technologies cat. no. A21121), type 2B (anti-mouse IgM Alexa555, Life Technologies cat. no. A21426), and laminin (anti-rat IgG Alexa647, Life Technologies cat. no. A21247). All antibodies were used at 1:150 for immunostaining.
Validation	These antibodies were validated for their use in mice by the supplier and by us via immunostaining on the basis of the correct staining pattern. All the antibodies here utilized have been extensively used in thousands of publications.

Animals and other research organisms

Policy information about studies involving animals; ARRIVE guidelines recommended for reporting animal research, and Sex and Gender in Research

Laboratory animals	Male C57BL/6J (The Jackson Laboratory, JAX#000664) mice were utilized at 5-6 months of age. Male BALB/c (The Jackson Laboratory, JAX#000651) mice were utilized at 3 months of age. CLXL7KO mice were previously described (Ghasemzadeh M, et al. The CXCR1/2 ligand NAP-2 promotes directed intravascular leukocyte migration through platelet thrombi. Blood 121, 4555-4566 (2013)). All mice were housed and handled in accordance with approved St. Jude Children's Research Hospital Institutional Animal Care and Use Committee protocols. Mice were housed in a ventilated rodent-housing system with a controlled temperature (22-23C), 40% humidity, 12-hour light/dark cycle, and given free access to food (standard chow diet) and water. Humane endpoints were not exceeded in any experiment. All euthanasia was performed with carbon dioxide in agreement with the recommendations of the Panel on Euthanasia of the American Veterinary Medical Association.
Wild animals	No wild animals were used in this study.
Reporting on sex	All experiments were done with male mice.
Field-collected samples	No field-collected samples were used in this study.
Ethics oversight	All mice were housed in the Animal Resource Center at St. Jude Children's Research Hospital, and handled in accordance with approved St. Jude Children’s Research Hospital Institutional Animal Care and Use Committee (IACUC). Additional accreditation of the Animal Resource Center at St. Jude Children's Research Hospital was provided by the Association for Assessment and Accreditation

of Laboratory Animal Care (AAALAC). Protocols employed in this study were approved by the Animal Care and Use Committee.

Note that full information on the approval of the study protocol must also be provided in the manuscript.
